# Lupus Erythematosus

**Published:** 1887-10

**Authors:** A. Ravogli

**Affiliations:** Cincinnati, O.


### *LUPUS ERYTHEMATOSUS.*

By Dr. A. RAVOGLI, of Cincinnati, O.

In his opening remarks, the author said that he would always prefer to treat lupus vulgaris than lupus erythematosus, so difficult was it to obtain satisfactory results. Kaposi had regarded the condition as a neoplasm. Hebra first described it as a seborrhœa, but modified the term by adding to it *congestiva*. All authors mention erythema and congestion as important features. Specimens were exhibited, which showed enlargement of the epithelial cells and hypertrophy of the papillæ in the stroma of the corium, and an infiltration of inflammatory cells in the meshes of the tissues, and some cells are seen between the fibres of connective tissue surrounding the hair-follicles. There is an increase in the number of connective-tissue corpuscles. The elastic fibres are swollen and enlarged, and the loculi, formed by the fibres of connective tissue, contain fluid. The blood-vessels are filled with blood. The condition is then one not of neoplasm, but is an inflammatory process. Each layer of the skin is affected. After a patch of lupus erythematosus has disappeared, there is left a thin, white, parchment-like condition of the skin, resembling a scar.

There is a true atrophy of the skin, caused by pressure, producing obliteration of the glands, which first take part in the hypertrophy. The process is then first a hypertrophy of the histological elements, followed by an atrophy.

The cause of the atrophy is found in the blood-vessels being closed and obliterated by pressure. The primary cause consists in an irritation and stimulation of the nerves, which increases the blood-supply and causes a disturbance in the biological activity of the cells.

The author has examined scales from three cases of lupus erythematosus. The epidermic cells were found enormously enlarged, and contained a large number of round bodies which he regarded as micrococci. They formed groups and colonies, and chemical tests appeared to confirm the opinion that they were such.

Sections of the skin were made, and showed the existence of the cocci in the papillary layer most abundant where exudation had taken place, and small colonies in the interior of the fibres and in the capillary blood-vessels.

These bodies answered to the test laid down by Friedlander for micro-organisms, and Dr. Ravogli does not doubt that micro-organisms exist in lupus erythematosus, not only upon the surface, but also within the structure of the skin. He has been unable to make any culture experiments. These organisms he regards as the cause of the disease, and instead of classing it as a neoplasm, thinks it should find its place among the infectious diseases.

The inflammatory symptoms and hypertrophy are explained by the irritation of the micrococci. The function of the sebaceous glands is increased by the irritation, hence the seborrhœa and the formation of the greasy scales. This explains lupus erythematosus discroides; in the form called aggregatus, the micrococci still better explain the condition.

As regards treatment, internal remedies are useless as a rule. The application of emplastrum hydrargyrum has been considered the best treatment. The benefit of this powerful microbe strengthens the theory. When the skin is destroyed with caustics, we succeed in destroying the cocci. When the sharp spoon was used to scrape the patches, or they were burned, the disease often returned in the neighborhood of the patch. Dr. Ravogli has obtained complete recovery in three cases of the disease by the use of ichthyol. Diminution in the secretion of the sebaceous glands is soon noticed. The reducing action of this agent is displayed on the underlying tissues. Ung. diachyl. hebræ goes well with ichthyol, (ten per cent.,) producing elevation of the epidermis and suppuration. The ichthyol is diminished to three per cent., and only slight scars result. He finishes treatment with ichthyol in collodion. Dr. Ravogli showed microscopic specimens to illustrate the pathological changes and the bodies which he regarded as cocci.

In the discussion, Dr. Knaggs, of England, asked if the reader considered the micrococci as causative, and whether the ichthyol destroyed them and the tissues, too, or acted as an antiseptic.

Dr. Ravogli replied in the affirmative. The ichthyol acted, he thought, by abstracting oxygen, upon which the life of the cocci depended.

Dr. Unna, of Hamburg, regarded the most important part of the paper that relating to micro-organisms, and he considered it the weak point as well. The cocci must be seen in all parts of the thickness of the skin, and, he believed, in the sweat-glands, too. He thought micro-organisms probably existed in the disease, and would some day be discovered, but could not regard the specimens presented as showing them, and hoped Dr. Ravogli would continue his investigations and make cultures. He asked if the sections were made deep enough to show the sweat-glands.

Dr. Ravogli said he had not paid special attention to these glands.

Dr. Unna was glad to hear of the good influence of ichthyol, never having used it alone in this disease, but with the addition of salicylic acid. He has found resorcin beneficial and most useful as a varnish or collodion dressing.

Dr. Thin, of London, said that the refractive particles seen under the microscope might be micro-organisms, and might be other bodies. More characteristic stainings would be necessary, and sections carried into the deeper parts, showing the same bodies, before they could be regarded as micro-organisms. Furthermore, cultures and inoculations would be necessary to prove the case. Dr. Thin spoke of several rare cases he had recently seen in England, which, from their peculiar appearance, had been given the name of the cock's-comb disease, as similar cases had not been observed there. The feature of the affection was a raised, bright-red, uneven surface, with abrupt margins. He had examined specimens of the tissue, and had found it identical with that of lupus erythematosus. There was a greater degree of vascularity than usual, the epidermis was raised, and a dense mass of small cells was found between the rete mucosum and strata lucidum. The affection was found very unsatisfactory as regarded treatment. He related one case of lupus erythematosus in which an inexplicable spontaneous cure had taken place while the patient was upon an exclusive milk diet, prescribed for another disease. He believed in a micro-organism, but thought it had not yet been found.

Dr. Klotz was glad to hear Dr. Ravogli say he avoided stronger and caustic remedies. He preferred salicylic acid combined with soap-plaster. He thought the discovery of micro-organisms would bring lupus erythematosus into closer relationship with lupus vulgaris, from which it had been too far alienated.

Dr. Zeisler, of Chicago, thought the micro-organism theory a very plausible one; still, it was only a theory. To prove them, it would be necessary to make cultures and inoculations. He did not regard the studies in this direction as entirely new, as Morrison, of Baltimore, had made similar investigations. Lupus erythematosus of the mucous membrane is an extremely rare condition. He had observed it in Vienna, in a case in which the mucous membrane of the mouth was affected. It presented a whitish condition of the membrane, the tissues were hardened, and it proved very obstinate under treatment. Caustic nitrate of silver was alone found to influence the condition. He agreed with Dr. Unna as to the value of resorcin, and that ichthyol had little effect. A ten per cent. solution of resorcin in collodion had acted well.

Dr. Robinson, the President, spoke especially in regard to the microscopic specimens. He agreed with Thin and Unna that in all probability a micro-organism exists, but he does not think that Dr. Ravogli has pursued proper methods, nor do the specimens show the bodies to be cocci. It is not clear that they are not extraneous matter. We must exercise great care in placing importance upon micro-organisms found upon the surface. He urged Dr. Ravogli to follow up his investigations, and thought something might come of it.

In closing, Dr. Ravogli said that he labored under difficulties in making his demonstrations at this time. He considered the test with caustic potash a convincing one, as it never attacked micro-organisms, but did attack everything else. He was glad to hear the opinion of so many that micro-organisms probably existed. He would not say positively at the present time that they were causative, but would wait until he had been able to make cultures, etc., as suggested.

